# Poor old pores—The challenge of making and maintaining nuclear pore complexes in aging

**DOI:** 10.1111/febs.15205

**Published:** 2020-01-23

**Authors:** Irina L. Rempel, Anton Steen, Liesbeth M. Veenhoff

**Affiliations:** ^1^ European Research Institute for the Biology of Ageing (ERIBA) University of Groningen University Medical Center Groningen The Netherlands

**Keywords:** chronological aging, nuclear pore complex assembly, nucleocytoplasmic transport, replicative aging

## Abstract

The nuclear pore complex (NPC) is the sole gateway to the nuclear interior, and its function is essential to all eukaryotic life. Controlling the functionality of NPCs is a tremendous challenge for cells. Firstly, NPCs are large structures, and their complex assembly does occasionally go awry. Secondly, once assembled, some components of the NPC persist for an extremely long time and, as a result, are susceptible to accumulate damage. Lastly, a significant proportion of the NPC is composed of intrinsically disordered proteins that are prone to aggregation. In this review, we summarize how the quality of NPCs is guarded in young cells and discuss the current knowledge on the fate of NPCs during normal aging in different tissues and organisms. We discuss the extent to which current data supports a hypothesis that NPCs are poorly maintained during aging of nondividing cells, while in dividing cells the main challenge is related to the assembly of new NPCs. Our survey of current knowledge points toward NPC quality control as an important node in aging of both dividing and nondividing cells. Here, the loss of protein homeostasis during aging is central and the NPC appears to both be impacted by, and to drive, this process.

## Graphical abstract

The nuclear pore complex (NPC) is the sole gateway to the nuclear interior, and its function is essential to all eukaryotic life. We summarize how the quality of NPCs is guarded in young cells and discuss the current knowledge on the fate of NPCs during normal aging in different tissues and organisms. Our survey points towards NPC quality control as an important node in aging of dividing and nondividing cells.

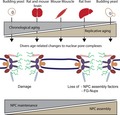

**Abbreviations**
EM: electron microscopy
ERC: extrachromosomal rDNA circles
ESCRT: endosomal sorting complexes required for transport
FG‐Nups: phenylalanine‐glycine‐rich nucleoporins
ID: intrinsically disordered
IDP: intrinsically disordered protein
INM: inner nuclear membrane
IQR: interquartile region
NE: nuclear envelope
NES: nuclear export signal
NLS: nuclear localization signal
NPC: nuclear pore complex
NTR: nuclear transport receptor
Nup: nucleoporin
ONM: outer nuclear membrane
Ran: Ras‐related nuclear protein
ROS: reactive oxygen species

## Introduction

One of the nine described universal hallmarks of aging is the loss of protein homeostasis 1. Loss of protein homeostasis can result from changes in the biogenesis, folding, trafficking, and degradation of proteins. The cell's ability to assemble and maintain functional protein complexes in aging is indeed compromised as changes in protein complex stoichiometry were highlighted as one of the prominent changes found across different aging organisms 2, 3. In baker's yeast, the nuclear pore complex (NPC; Box 1) is among the most substoichiometric complexes in replicative aging cells 2. NPCs are the conserved gates to the nuclear interior and essential to all life. A prominent function of NPCs is to facilitate the transport of macromolecules from the cytoplasm to the nucleus and vice versa. The proteins of the core scaffold of NPCs are extremely long‐lived in *Caenorhabditis elegans* and in rat neurons 4, 5, 6, and also in replicative aging yeast cells, the scaffold components are long‐lived 7. Additionally, the transcription of those core scaffold proteins is downregulated in differentiated cells 4, suggesting that there is little synthesis of new NPCs in differentiated cells. Phenylalanine‐Glycine‐rich nucleoporins (FG‐Nups), on the other hand, often turn over quickly, especially in metazoan cells 4, 6, 8 and to a lesser extent in yeast 7, 9, 10. These differences in turnover rates might make the NPC particularly sensitive to changes in protein homeostasis in aging. The assembly of new NPCs is complex and heavily dependent on the presence of the proper amounts of NPC components and assembly factors. Altogether, the biochemical properties of NPCs set them at risk for age‐related decline and, indeed, several studies addressed age‐related changes of the NPC, and/or nucleocytoplasmic transport in aging 4, 11, 12, 13, 14.

Box 1 **The structure and function of the nuclear pore complex at a glance**

**Figure 1:**
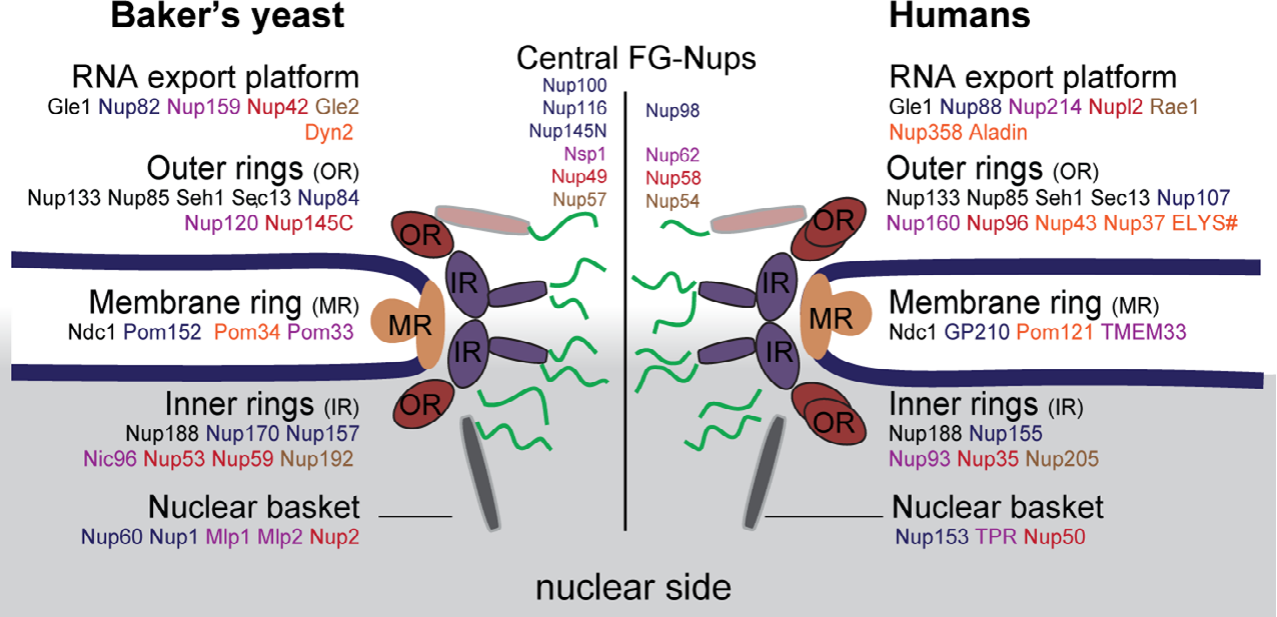
Cartoon representation, adapted from 17, of the main structural features of NPCs and conserved proteins between baker's yeast (*Saccharomyces cerevisiae*) and humans. The main structural components (RNA export platform, outer rings, membrane ring, inner rings, and nuclear basket, as well as the eight‐fold rotational symmetry of these structures) are conserved from yeast to humans 30. The outer rings are composed of Y‐shaped complexes; yeast NPCs have a total of two outer rings, one on the cytoplasmic and one on the nuclear side respectively. Humans have a total of four outer rings, two on the cytoplasmic side and two on the nuclear side. Human NPCs are considerably larger in their dimensions and their molecular mass (not shown) than yeast NPCs. The position of Nups within the NPC and their stoichiometry are best known in the yeast NPC (e.g., in 17, 31). Pom33, Nup2, Gle2, Dyn2 are dynamic or have prominent alternative locations or functions but also several bona fine Nups have roles away from the NPC. Most notably, the outer ring proteins Sec13 and Seh1 are also components of the COPII vesicle coat, and the membrane protein Ndc1 is also at the spindle pole body. Names of functional homologs within a structural component have the same color (e.g., the inner ring proteins Nic96‐Nup93, or Nup53‐Nup59‐Nup35). Nups that are unique for either yeast or human are written in orange font. #ELYS is only present in the outer rings at the nuclear side.

The NPC structure, recently reviewed in 15, is made of ~ 30 different proteins, called Nucleoporins or Nups, that are present in multiple copies (Fig. 1). Each NPC is composed of eight spokes, which are arranged to form a cylindrical structure embedded in the nuclear envelope (NE) membranes. Each spoke is interconnected by flexible linker elements that probably give the NPC strength and flexibility at the same time 16, 17, 18. The core of the NPC is organized by stably folded proteins in symmetric inner and outer rings and is anchored to the NE by transmembrane proteins. The outer rings are mainly formed by a protein complex called the Y‐shaped complex. Attached to the cytoplasmic side of the symmetric core is a structure called the RNA export platform (also called ‘cytoplasmic filaments’). Attached to the nuclear side of the symmetric core is the nuclear basket, which is involved in nucleocytoplasmic transport, RNA processing, and RNA export, but also serves as a multifunctional platform for various nuclear processes (e.g., transcriptional and chromatin organization). Both structures, the RNA export platform and the nuclear basket, are involved in RNA processing and RNA export. In the center of the core scaffold are intrinsically disordered (ID) proteins that form the selective barrier of the NPC, the FG‐Nups. A decrease in the concentration of FG‐Nups in the center of the NPC compromises the permeability barrier and active transport rates of the NPC 19, 20, 21.

Rapid and energy‐dependent transport across the NE is facilitated by the NPC and several transport factors (reviewed in 22, 23, 24, 25, 26). The energy‐dependent translocation of macromolecules between the nucleus and the cytoplasm is facilitated by nuclear transport receptors (NTRs) and the GTP binding Ras‐related nuclear protein (Ran). There are 17 different NTRs known in yeast 27, and about 30 NTRs are known in humans 28. For many proteins that are transported either to the nucleus or the cytoplasm, their respective NTRs remain to be identified. In addition, other transport mechanisms can be utilized for transport; for example, the energy for nuclear export of most mRNA is provided by ATP hydrolysis on DEAD‐box helicases 25.

Proteins that require nuclear import encode a nuclear localization signal (NLS) and are recognized in the cytoplasm by NTRs called importins. Proteins that require nuclear export encode a nuclear export signal (NES) and are recognized in the nucleoplasm by NTRs called exportins. For proteins without an NLS/NES targeting signal, the NPC acts as a diffusion barrier. Generally, it can be stated that the size and surface properties of a molecule determine how easily a molecule can diffuse between nucleus and cytoplasm 19, 21, 29.

In this review, we compare the age‐related changes in NPCs found in different model organisms and different tissues and we further discriminate between aging in nondividing cells (chronological aging) and aging in dividing cells (replicative aging). For age‐related diseases caused by mutations to the NPC, or its supporting machinery, we refer to recent reviews 32, 33, 34. We compare specifically age‐related changes in the abundances of the components of the NPC (Nucleoporins or Nups) as found in different proteome studies. The changes in Nup abundances observed during replicative and chronological aging of bakers’ yeast cells are distinct, indicating that dividing and nondividing cells potentially face different challenges in NPC assembly and maintenance, respectively. Based on our analysis of Nups in aging, we discuss to what extent replicative and chronological aging in baker's yeast are suitable models for the aging of tissues with faster turnover rates, like liver, or long‐lived tissues, like the brain. We further speculate on what could be causal for the age‐related changes at the NPC. For this, we summarize the current knowledge of the mechanisms of NPC assembly and maintenance and point out open questions. In the last part of this review, we summarize how NPCs could contribute to the hallmarks of aging.

## On the search for common age‐related changes in NPCs of different cell types

### General considerations for the comparison of aging profiles

**Figure 2:**
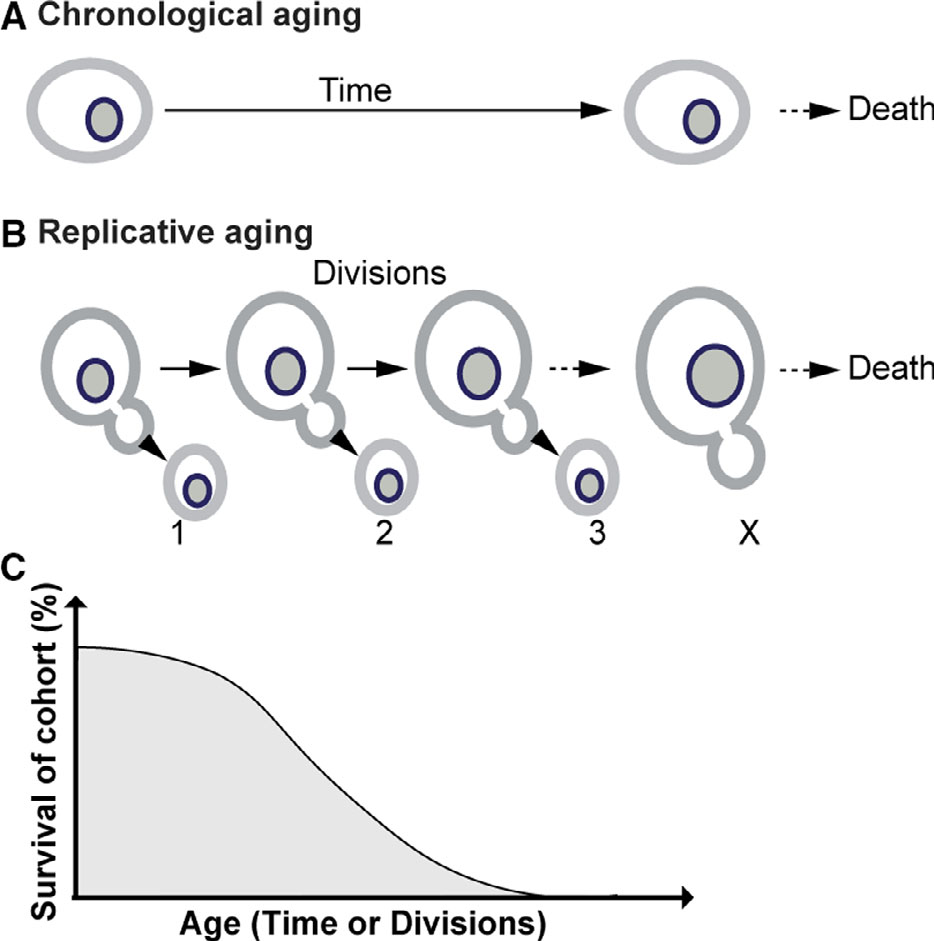
Schematic representations of aging on the population and the single‐cell level. (A) Cartoon of a chronological aging baker's yeast cell. Chronological aging is the kind of aging that nondividing cells experience in time before they die of old age. Chronological aging is induced through the depletion of nutrients. Commonly used protocols to achieve nutrient depletion include growing a cell culture to a stationary phase or transferring an exponential culture to water 52. Acidification of the medium has been a cofounding factor in some chronological aging studies 53. Moreover, prolonged starvation is a stress that postmitotic cells in higher eukaryotes do not experience, and this should be considered when translating results from chronological aging studies in *Saccharomyces cerevisiae* to higher eukaryotes. A more involved way to induce chronological aging, which overcomes the limitation of severe nutrient depletion in yeast, is to provide the cells with just too little nutrients to divide, called near‐zero growth and which is performed in a retentostat 40. (B) Cartoon of a replicative aging baker's yeast cell. Dividing cells age with each completed division, therefore their age is measured in the number of completed divisions. Replicative aging in baker's yeast is triggered by the asymmetric retention of aging factors in the mother, which causes the mother to age while the daughter cell is born young. Age‐induced damage can occur in the form of damaged and nonfunctional organelles, ERCs, asymmetrically retained transmembrane proteins and protein aggregates. (C) Cartoon of a typical survival curve of a cohort of aging cells or organisms. Aging is associated with an increased risk of mortality and there is intrinsic variation in the lifespan of individual cells or organisms within one cohort. As aging occurs at very different timescales, cross‐species comparisons of changes associated with aging, as well as comparisons between chronological and replicative aging cells can be based on the survival of the cohort.

In general, there is a large degree of conservation of age‐associated changes across different eukaryotic species 1, 35, 36, 37, 38, 39. To investigate whether NPCs change in similar ways in aging cells and tissues from different species, we extracted data on the abundance of Nups from six published proteome datasets, derived from aging budding yeast 2, 40, rat 3, and mouse 41. The studies using baker's yeast addressed proteome changes that occur during aging in nondividing cells, chronological aging (Fig. 2A) 40, and in dividing cells, replicative aging (Fig. 2B) 2. The studies with rats focused on age‐related changes in brain and liver tissues, and those with mice studied age‐related changes in brain and muscle tissues. All three tissues are primarily composed of postmitotic cells. The majority of brain cells are extremely long‐lived, and the brain has a very limited regenerative capacity 42. The liver, on the other hand, has high regenerative capacity, and liver cells are turned over regularly 43, 44. Skeletal muscle is predominantly composed of polynucleated muscle fibers that can regenerate when injured 45.

**Figure 3:**
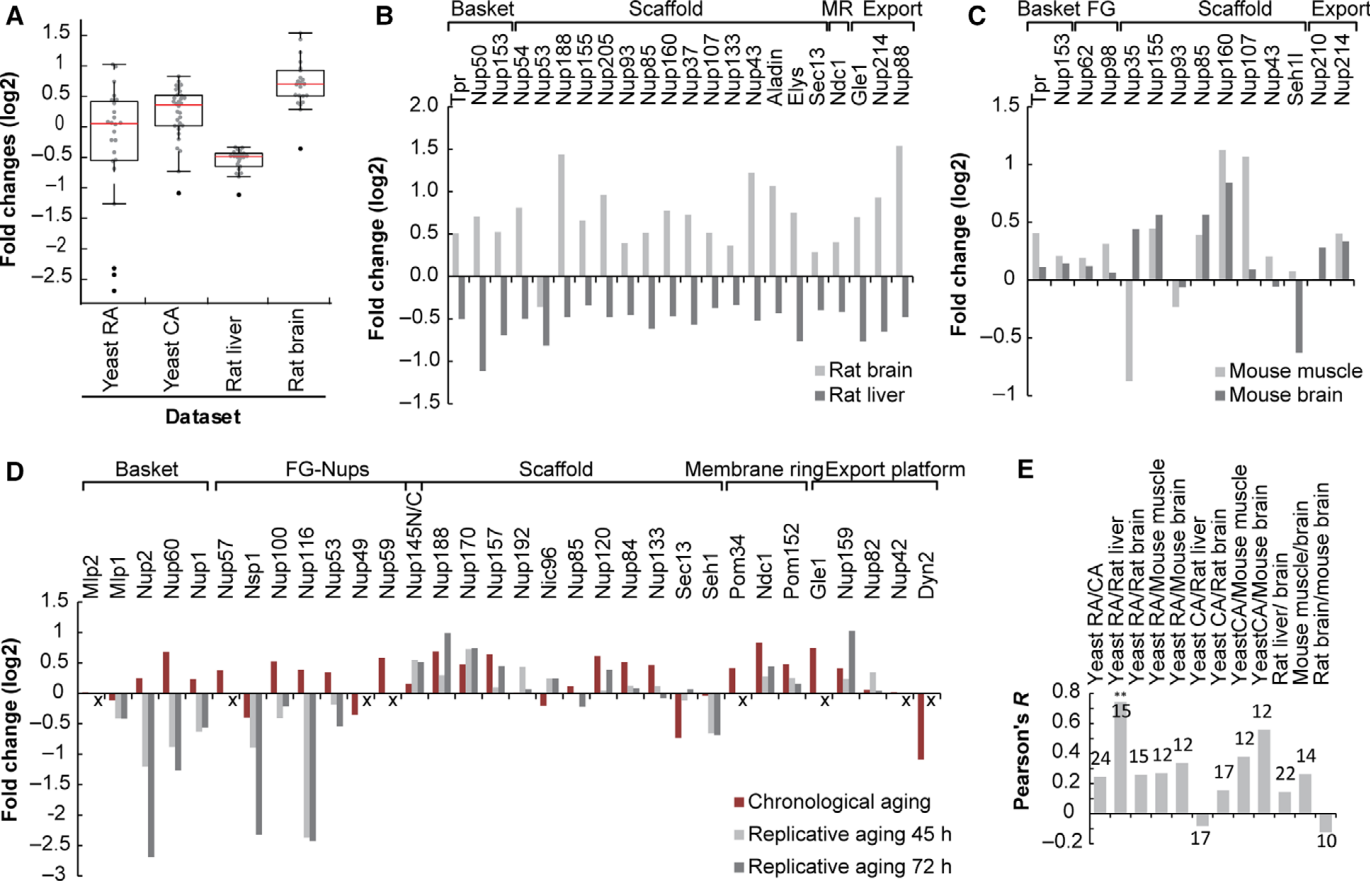
Loss of overall NPC stoichiometry in different aging model systems: budding yeast 2, 40, rat 3, and mouse 41. (A) Fold changes in abundance of Nups from different aging proteome data sets, comparing the changes in aged mice and rats (24 months, 50–70% viability) and replicative aging yeast (Yeast RA; 72 h, 55% viability) and chronological aging yeast (Yeast CA; 60% viability). Fold changes of individual Nups from each dataset are plotted as gray dots and are overlaid with a boxplot. 24, 32, 22, and 22 Nups were measured in the yeast RA, yeast CA, rate liver, and rat brain samples, respectively. The red line depicts the median fold change and the boundaries of the box mark the 25th and 75th percentile of fold changes in the dataset. The height of the box (IQR) is influenced by the spread within the middle 50% of the data and is a robust measure of dispersion, that is, insensitive to outliers. The whiskers extend to the data points which are not considered outliers, which are shown as black dots. (B) Comparison of age‐related changes in Nup abundance of rat brain and rat liver. None of the individual changes were reported significant according to the criteria of the authors 3. (C) Comparison of age‐related changes in Nup abundance of mouse muscle and mouse brain. Only the change in Nup107 in muscle was reported significant according to the criteria of the authors 41. (D) Comparison of age‐related changes in Nup abundance of yeast chronological at a time point of ~ 60% population viability and yeast replicative aging at time points representing ~ 75 and 55% viability (45 h and 72 h). The replicative aging proteome does not include information on the abundance of Mlp2, Nup57, Nup49, Nup59, Gle1, Nup42, and Dyn2, represented by an ‘*x*’ on the *x*‐axes. In the chronological aging datasets, the changes for Nsp1, Pom152, Ndc1, Nup57, Nup170, Nup188, Nup60 Nup157, Sec13, Dyn2, Nup60 were reported significant according to the criteria of the authors 40; the data for the first replicate are shown. For the replicative aging dataset, no fold change statistics were reported but rather a noise threshold was applied to the time series datasets using the coefficient of variation between replicates with a cutoff of 0.3, retaining only the most reproducible data 2. The pronounced decrease in abundance of Sec13 and Dyn2 during chronological aging may not reflect changes at the NPC but rather reflect their roles cell division 61, 62. (E) Pearson correlations of pairwise comparisons of changes in Nup abundances in samples from different aging proteomes. The number on top of the bars indicates the sample size of Nups that were used for the comparison. ** indicates a significant correlation with *P* < 0.01. The correlation coefficient ranges from −1 to 1. Values close to −1 indicate negative, and values close to 1 indicate positive linear relationships between two samples. Values close to 0 indicate that there is no linear relationship between two samples.

The comparison of aging between different model organisms is not trivial for four reasons. First, replicative aging and chronological aging are measured in different units (number of divisions and time in nondividing state) and different model organisms have different maximal lifespans. Therefore, a fair way to compare the different aging trajectories is to align them based on the average survival of the population (Fig. 2C). For the specific proteome studies of aging yeast, mouse and rats 2, 3, 40, 41 that we compare in Fig. 3, we estimate, that the viability of the yeast replicative aging population is about 55% at the last time point measured (72 h, ~ 24 divisions) 2, the viability of the yeast chronologically aged population is ~ 60% (after 21 days) 46, and the mice and rat should have a survival of ~ 50–70% at the latest age time points (24 months) 3, 47, 48. Rat and mouse strains used in the mentioned studies have similar lifespans. Both aged samples were analyzed 24 months after birth. However, the young mouse sample was taken 3 months 41, and the rat sample was taken 6 months after birth 3. Second, aging is a highly individual process and analyzing only two or three animals per sample will not cover the full spectrum of aging changes in a population. Third, the age‐related changes in protein abundance as measured in a tissue might be caused by age‐related changes in the abundances of specific cell types that compose a tissue rather than reflecting changes in NPCs of one cell type 49, 50, 51. Lastly, different methods were used to prefractionate the mouse and rat samples, to do the mass spectrometry, and to analyze the data. Most significantly, the mouse and rat proteomes are from fractionated tissue samples, and specifically, the nuclear fraction was analyzed, while the yeast samples contain whole‐cell extracts. Even taking these limitations into account, there are interesting similarities and differences in the aging trajectories of NPCs that we will highlight in the following sections.

### Loss of stoichiometry of NPC components

Loss of protein complex stoichiometry, in general, is a conserved phenotype of aging 2, 3, and NPCs are among the most substoichiometric protein complexes in replicative aged yeast cells 2. The rat liver and brain and the yeast replicative aging and chronological aging proteome datasets have a reasonable coverage of the NPC components (22, 32, and 24 Nups are reported in the rat, yeast chronological aging, and replicative aging datasets, respectively), so that we can assess whether the NPCs become similarly substoichiometric in these aging cells and tissues.

The comparison of the changes in Nup abundances in the proteome datasets shows that loss of Nup stoichiometry is most pronounced in yeast replicative aging cells, followed by yeast chronological aging cells and aging rat brain tissue. In the rat liver tissue, the stoichiometry of NPC proteins is most stable in aging (Fig. 3A). While the datasets are not suitable to interpret changes of individual Nups, the overall impression is that the loss of stoichiometry in NPCs is driven by different changes in the different cells and tissues (Fig. 3B,C). For example, a pairwise comparison of changes in the abundance of Nups during yeast replicative and chronological aging shows that the majority of Nups building the NPC scaffold and membrane ring show similar increases in abundance during replicative and chronological aging (Fig. 3D). In contrast, the strong loss of several nuclear and central FG‐Nups is specific to replicative aging. Also, comparing the proteomes of liver and brain samples, a major conclusion of Ori *et al*. 3 was that the age‐related changes in protein abundance are tissue‐specific in general, and this seems to hold true for the Nups as well (Fig. 3A,B).

Overall, the comparison of the changes in Nup abundances in aging mice, rats, and replicative and chronological aging yeast cells reveals that the NPC becomes substoichiometric to different extents, most strongly so in replicative aging yeasts, and that the specific changes in Nup abundances are distinct in each dataset.

### Yeast as a model system to study the aging of NPCs in higher eukaryotes

Yeast has been a powerful model in aging research for many practical reasons 35, 36, 54, 55. Specifically, for the proteomics studies discussed in this review, the advantages are that the coverage of the Nups is higher in yeast due to the relative simplicity of the proteome. Also, the methods for cultivation of aging yeast cells 2, 40 allow the assessment of many time points in the aging trajectory (12 in the case of Janssens *et al*. 2), which increases the confidence of observed changes. Indeed, the comparison of the replicative aging proteome with more targeted studies on the yeast NPC in replicative aging (Table 1, Janssens *et al*. 2, Rempel *et al*. 11, and Lord *et al*. 13) is overall consistent. Such a level of consistency is currently not seen for the analysis of rodent brain samples. It is, however, important to address the question to what extent yeast is suitable for aging studies of the NPC specifically.

**Table 1:** Key findings related to NPC composition and transport function in aging. ↓ indicates a decrease in protein abundance, while – indicates no change in protein abundance was detected. The font colors indicates the location of the protein in the NPC, see Fig. 1: central FG‐Nups (green), inner ring (purple), outer ring (dark red), nuclear basket (gray), and RNA export platform (pink).

Study	Model organism	Nup protein abundances		Nup transcripts abundances	Transport phenotype
D'Angelo *et al*., 2009 4	*Caenorhabditis elegans* rat brain (24–28 month)	Nup93↓ FG‐Nups↓ Nup107‐	Nup93, Nup153 carbonylated	Downregulation of scaffold Nups in differentiated cells	Increased passive permeability of the NE of isolated nuclei and intranuclear tubulin bIII *in vivo*
Kim *et al*., 2010 12	Senescent human fibroblast (> 66 doublings)	Nup88↓ Nup107↓ Nup155↓	Nup50↓	Decreased transcript levels of Nups, but also importin α/ß and Ran system	Fewer NPCs, decreased nucleocytoplasmic transport, unresponsive to cell stimuli
Lord *et al*., 2015 13	*Saccharomyces cerevisiae,* replicative aging (6–9 divisions)	Nup100– Nup53– Nup116↓	Nsp1↓	Stable transcript levels of Nup100, nup53, Nup116 and Nsp1	Decreased nuclear accumulation of different NLS‐GFP reporters at steady state
Rempel *et al*., 2019 11	*Saccharomyces cerevisiae* replicative aging (multiple time points)	Nup100↓ Nup133– Nup116↓ Nup2↓ Nup120 ‐			Increased nuclear accumulation of NLS‐GFP reporters and Rcc1 at steady state and decreases exclusion of NES‐GFP reporters, decreased transport dynamics of Msn2 shuttling
Janssens *et al*., 2015 2	*Saccharomyces cerevisiae* replicative aging (12 time points)	Nup116↓↓ Nup2↓↓ Nsp1↓↓ Nup100↓ Nup60↓	Nup1↓ Mlp1↓ Gle2↓ 15 others‐	Stable transcript levels for all Nups	

General complications in the comparisons of aging yeast cells with aging tissues in multicellular organisms are that aging in yeast occurs on much shorter timescales, and also that aging experiments with yeast are performed such that they report only replicative or only chronological aging. This is not the case in multicellular organisms, where cells are destined to carry aspects of both chronological and replicative aging. For example, blood hematopoietic stem cells may stay quiescent for years before starting their replicative lifespan 56 and, vice versa, postmitotic cells were derived from stem cells that themselves experience aspects of replicative aging. The most direct comparison between the replicative and chronological aging regimes in yeast would be on the cellular level, namely with asymmetrically dividing mitotic cells, like stem cells, and postmitotic cells, like neurons 54, 55. With respect to the comparison in replicative aging, the immortal yeast daughter lineage should be compared to the self‐renewing stem cell lineage. The yeast mother cells and the differentiated cells both are both mortal and retain damaged components 54, 57, 58, 59, 60.

These general considerations of how yeast replicative and chronological aging may relate to aging in rodent tissues are indeed somewhat reflected in the analysis of age‐related changes of NPC components as follows: The age‐related changes in Nup abundances in yeast replicative aging correlate with those in aged rat liver, where the shared loss of the basket Nups is the main contributor to this correlation being significant (Fig. 3E). The changes in yeast chronological aging do not reach significance with any of the other datasets but best correlate with those in mouse brain (Fig. 3E). Overall, the age‐related changes in Nups in replicative and chronological aging yeast models bear only a weak resemblance to the current datasets describing tissues that are generally considered to be aging replicative (liver) and chronological (brain).

## Potential causes for age‐related changes of NPCs: assembly and maintenance of NPCs

### Unique biochemical properties set NPCs at risk in aging and aggregation pathologies

To understand what could be the cause for the distinct changes in the NPCs in different aging regimes and cell types, we discuss how the NPC may be facing different challenges. Based on the fact that the transcript levels of many Nups are stable in aging (see Table 1), we discuss mostly posttranscriptional aspects of NPC stability, namely NPC assembly and NPC maintenance. First, the assembly of NPCs is a complex process that occurs through two different mechanisms recently reviewed in 15. In higher eukaryotes, NPCs and the NE disassemble at the start of mitosis. Consequently, all NPCs need to be reassembled into the reforming NE after each division (postmitotic assembly). Organisms with closed mitosis such as budding yeast, and interphase cells from higher eukaryotes, assemble NPCs into the intact NE (*de novo* or interphase assembly) 63. Second, once NPCs are functionally formed their maintenance is a challenge. Parts of the NPC are long‐lived and thus susceptible to the accumulation of damage over time. We discuss the importance of oxidative damage in this context. Third, the ID FG‐Nups readily self‐associate and aggregate 64, 65, 66 and we briefly discuss the recently established connection between NPCs and aggregation pathologies. Fourth, we discuss the current knowledge about the mechanism for quality control of NPCs, an exciting new area of research 67, 68, 69.

### Two mechanisms of NPC assembly: interphase assembly

Interphase assembly occurs into the intact NE; hence, it is challenging to study as it requires detecting single NPC assembly sites amidst the many fully formed NPCs 70, 71, 72. Scanning electron microscopy (EM) studies using *Xenopus* egg extract reported that that assembly begins with the formation and stabilization of a hole (pore) in the NE 72. A more recent study showed transmission EM images of NPC interphase assembly intermediates in mammalian cell lines 71. These revealed that interphase assembly occurs through the evagination of the inner nuclear membrane (INM), which further deforms until it fuses with the flat outer nuclear membrane (ONM). The site of the deformed membrane is a mushroom‐shaped, electron‐dense mass of growing size 71.

It remains to be determined, in which order the Nups exactly assemble, how the evagination of the INM is achieved, which non‐NPC components might be involved in stabilizing the assembly intermediates and which mechanism ultimately fuses INM and ONM. The transmembrane proteins Sun1 and Pom121 establish where a new NPC is assembled, and Pom121 is part of the early assembly intermediate 73. Nup153 and Nup53 are associated with these early assembly intermediates 71. The outer ring complexes probably join the assembly later, potentially after the fusion of INM and ONM 73. The RNA export complex also joins only later in NPC assembly 71, and assembly problems are often associated with mislocalization of proteins of this complex, while other Nups still localize to the NE. Interphase assembly factors Heh1, Heh2, Apq12, Brl1, Brr6, Rtn1, Rtn2, and Yop1 have been studied in baker's yeast 69, 74, 75 and assist in the assembly process including the membrane fusion, but it is not known in which steps of NPC assembly these proteins are acting. Many NPCs are assembled during interphase, which is regulated by the protein levels and phosphorylation state of the basket protein Tpr. Extracellular signal‐regulated kinase phosphorylates Tpr at the NPC, and while associated with the NPC, it also phosphorylates Nup153. Phosphorylation of Nup153 then prevents Nup153's association with the outer ring complex and blocks further NPC assembly 76, 77. Cancer cells often have more NPCs 78, and there is evidence suggesting that NPC numbers also change in aging 3, 54. Interestingly, both yeast aging datasets and the rat liver proteomes show that the levels of the Mlp1/Tpr proteins (and the other basket Nups human Nup50 and yeast Nup2) decrease relative to the other Nups. As the absence of Tpr is reported to increase NPC numbers 77, this could indicate that more NPCs are assembled in aging. This is not in line with the decline in NPC number reported in rat liver 3, but it is in line with the overall increase in Nup levels at the NE in yeast 11, 54.

The effects of having too many or too few NPCs are not well understood. Increasing numbers of NPCs at the NE will result in an increase in passive diffusion of molecules over the NE. The rate‐limiting step for NTR‐dependent transport is the formation of a complex between the NTR and its cargo: It takes time for the NTR and cargo to find each other in the crowded cytosol, while the actual translocation through the NPC is fast 79, 80, 81. We thus speculate that a moderate decrease in NPC numbers will not affect energy‐dependent transport. However, when NPC numbers are greatly reduced (~ 50%), the number of NPCs becomes rate‐limiting and energy‐dependent transport rates were shown to drop 82.

In baker's yeast, some of the proteins that assist and monitor the assembly of NPCs are identified, but their mechanism of action is only beginning to be understood. Key players thus far identified are the INM proteins Heh1 and Heh2, the NE‐specific endosomal sorting complexes required for transport (ESCRT)‐III adaptor Chm7, and the ESCRT‐III/Vps4 system (see Box 2) 68, 69, 83. Interference with this quality control mechanism results in the accumulation of misassembled NPCs 69. The proteins that guard NPC assembly in baker's yeast are conserved in higher eukaryotes where Chmp7 and the ESCRT‐III system additionally have the important function of resealing the NE at the end of mitosis 84. In baker's yeast, several proteins that assist and control the quality of NPC assembly decrease in abundance during aging, and there are indications that aged yeast cells experience problems with NPC assembly (more details in Box 3).

Box 2 **NPC quality control in baker's yeast**

Pioneering work from the Lusk laboratory has revealed the first data on how cells deal with defective NPC intermediates. Although it is early days, the data support the following model for sealing off defective NPCs and defective NPC intermediates: Their recognition is achieved through the loss of spatial separation between the NE‐specific ESCRT adaptor, Chm7, and the INM protein Heh1 83. When Chm7 reaches the nucleus, it binds to Heh1/Heh2, which allows sealing off the defective NPC. Additionally, the interaction allows the ESCRT machinery to assemble at the site of the defective NPC 68, 69. The data further support that successful clearance of the sealed off NPC from the NE depends on ESCRT‐III proteins Vps2, Vps24, and Snf7, where Snf7 binds directly to Heh2 and Chm7. Vps20 does not seem to be part of the nuclear ESCRT‐III complex 69, despite it being characterized as part of the core ESCRT‐III complex 85.

Based on the above, and knowledge of the ESCRT‐III membrane scission system, a possible scenario is that upon Chm7 binding, Snf7 assembles into a polymer, which is capped by Vps24. Vps24 then recruits Vps2 to the complex and promotes the assembly of a Vps4 hexamer at the site of the misassembled NPC. Vps4 disassembles the ESCRT‐III complex while hydrolyzing ATP. What kind of membrane remodeling is needed to remove misassembled NPCs from the NE is currently still unknown 86, but membrane remodeling is normally achieved through the disassembly of the Snf7 filament by Vps4 85. Surprisingly, this process seems to be independent of the Vps4 cofactor, Vta1, as no synthetic genetic interactions between Vta1 and various tested Nups are found 69 raising the question how effectively the filament are disassembled 87. Concerning the final degradation of the misassembled NPC, there is evidence that the proteasome and not the vacuolar peptidases play a role in the degradation of Nup85 69.

### Two mechanisms of NPC assembly: Postmitotic assembly

Our knowledge concerning NPC assembly is derived from different model organisms. The order in which Nups assemble during postmitotic assembly has been studied in Xenopus egg extract and fixed human cells at different cell‐cycle stages 88, 89, 90, 91, 92. Additional structural insight comes from *in vivo* studies of rat and human cell lines 93, 94, 95, 96. Postmitotic assembly of NPCs happens in organisms with open mitosis upon mitotic exit, in telophase. All NPCs reassemble simultaneously into small openings at the reforming NE envelope during a short timeframe of only 5 min 93, 96. The initiation of NPC assembly starts in late anaphase with the association of ELYS with decondensing chromatin at the nuclear periphery 92. The Ran‐GTP dependent release of Nups from importin ß 97 allows the assembly of outer ring complexes, which are then bound to chromatin by ELYS at NPC assembly sites 92. At this stage, Ndc1 and Pom121 are recruited to the assembly site to establish contact with the reforming NE. Subsequently, the Nup93‐complex assembles at the prepore, which is followed by the Nup62 complex and several (other) FG‐Nups 98. The last components to join the reassembled NPCs are the parts of the nuclear basket and the assembly of the RNA export platform (cytoplasmic filaments) 89, 93.

It was earlier noted that in dividing cells NPCs might be renewed during reassembly after each division 4, 99. Rat liver cells from old rats were characterized by decreased levels of several Nups suggesting an overall reduction of NPCs. One explanation for the reduced number of NPCs in the liver of aged rats is, that the postmitotic assembly of NPCs might be an opportunity for cells to clear up substoichiometric protein complexes, in line with this interpretation is also the low interquartile region (IQR) of the aged rat liver sample (Fig. 3). Such an opportunity does not apply to the aging baker's yeast cell, where the NE and NPCs remain intact during the entire division. Indeed, there is evidence in baker's yeast, that replicative aged cells have problems to correctly assemble NPCs, and potentially also to clear misassembled NPCs from the NE (Box 3) 11.

Box 3 **NPCs in replicative aging—a yeast perspective**

**Figure 4:**
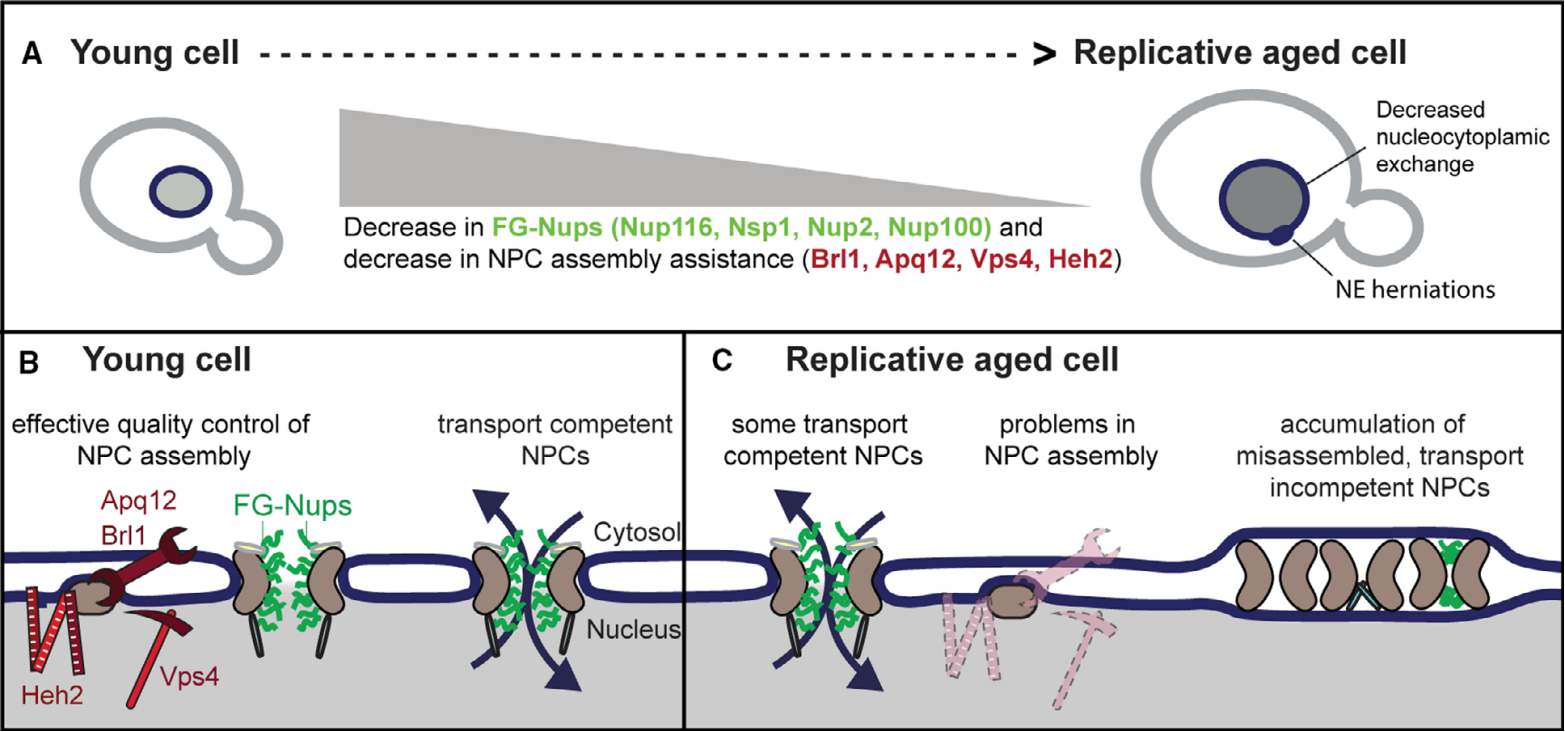
Model of age‐related changes at the NPC during yeast replicative aging 11. (A) Summary of nuclear transport‐related changes occurring in replicative aging baker's yeast cell, as measured in 2, 11, 13: (a) The abundance of indicated FG‐Nups and proteins assisting in NPC assembly declines with replicative aging, (b) aged cells more frequently have NE herniations, and (c) nucleocytoplasmic exchange is decreased in aged cells. (B) Schematic representation of NPC assembly and nuclear transport dynamics in young cells based on 68, 69, 83. (C) Model: In aged cells, the decrease in the abundance of several proteins that assist in NPC assembly (indicated by faded cartoons) causes the accumulation of misassembled NPCs in aged mother cells. Misassembled NPCs are covered with membrane and do not participate in nucleocytoplasmic exchange. In other words, the overall number of transport competent NPCs is reduced in replicative aged mother cells.

Several studies in baker's yeast showed that NPC components are present in substoichiometric amounts in replicative aged cells. More specifically, a strong decrease in abundance (at the whole‐cell level and at the NE) was observed for the FG‐Nups Nup116, Nsp1, and nuclear basket protein Nup2 2, 11, 13 (Fig. 4A). On the single‐cell level, the age‐dependent decrease in abundance of Nup116 and Nup100 at the NE was correlated to the lifespan of the cell, where decreased levels correlated with less remaining lifespan 11. The deletion of Nup100 was previously described to extend the replicative lifespan, through an increase in cellular Gcn4 levels 13, 100, illustrating that the full deletion of a gene in young cells can have a very different outcome than the age‐dependent decrease in abundance in old cells. Overall, the changes in NPC stoichiometry during replicative aging are well documented and changes are consistent between different studies.

Studies of the NPC assembly and quality control machinery show that the aging cells lose several components, which normally ensure that NPCs are assembled correctly, or broken down if misassembled. Namely, Apq12, Brl1, Heh2, and Vps4 showed decreased abundances in aging 11. How these proteins influence the assembly of NPCs is still not fully understood (Fig. 4B). Previous studies suggested that Apq12, Brl1, and Brr6 are primarily involved in lipid homeostasis 101, 102 and changes in lipid composition could cause NPC assembly defects. A more recent study reports a more direct involvement in NPC assembly 75. The decreased abundance of Apq12, Brl1, Heh2 and Vps4 thus suggests that NPCs in old cells misassemble more frequently and are less effectively cleared from the NE (Fig. 4C). In aged cells, Chm7 foci, representing NPC assembly problems, indeed appear almost three times as frequently as in young cells 11. Misassembled NPCs and those that have extrachromosomal rDNA circles (ERCs) tethered to their scaffold are asymmetrically retained by the mother cell 54, 69, 103. Apq12, Brl1, and Brr6 are conserved proteins in eukaryotes with closed mitosis. Although these proteins are not conserved on the sequence level in higher eukaryotes, functional homologues may exist 86, 104.

It is unclear whether the altered Nup levels in replicative aged cells are a cause or a consequence of misassembled NPCs, or even of problems with NPC maintenance. The average Nup abundance at the NE, as measured using imaging methods, for example, represents fully assembled functional NPCs, as well as potentially damaged NPCs and/or misassembled NPCs with altered Nup composition. The loss of the FG‐Nups (Nsp1, Nup116, and Nup2) may thus reflect that there is a subset of misassembled NPCs that partially or fully lack those FG‐Nups. These NPCs likely do not contribute to transport, as NPCs lacking these specific Nups would increase the passive permeability of the NE, and no such change is observed in aging 11. Instead, these misassembled NPCs are likely covered by membranes making them transport incompetent. The NE is often herniated at the site of membrane‐covered NPCs. Indeed, herniations have been observed in specific mutants, for example, mutants lacking Nup116 105, and they are enriched in aged cells 11.

The changes in the populations of NPCs at the NE have functional consequences for the cell that are not fully understood. Aged cells show increased nuclear compartmentalization of GFP‐NLS and GFP‐NES reporter proteins, as well as an increase in nuclear localization of Rcc1‐GFP 11. Consistently, the shuttling transcription factor Msn2 shows decreased shuttling dynamics during aging and the decrease in shuttling is correlated to lifespan 11. Lord *et al*., 2, 11, 13 report loss of nuclear localization of GFP based reporters with different NLSs in aging. Apart from technical differences (different yeast strains and methods of aging), we speculate that the results may also reflect different stages of the aging process. For example, a moderate reduction of NPCs early in life would cause increased nuclear compartmentalization, as this may primarily decrease passive diffusion of the reporter proteins over the NE. Only late in life, the number of NPCs may become rate‐limiting for active transport. Several studies indicate that changes in the permeability barrier and the steady‐state localization of proteins are quite well tolerated by the cell 13, 20. In contrast, even moderate changes in nuclear transport dynamics correlated to the remaining lifespan, suggesting that changes in dynamics are detrimental for the cell as it interferes with their ability to react to its constantly changing environment 11.

### Mechanisms of NPC maintenance

In metazoans, the Nups have vastly different turnover times. Among the group of particularly long‐lived Nups are Nup93, Nup96, Nup107, and Nup205, while Nup133 has intermediate turnover times, and Pom121, as well as FG‐Nups, are replaced regularly 5, 6, 67. The first insights into NPC maintenance in postmitotic cells were only published recently 67. This study revealed two different mechanisms of NPC maintenance. Quiescent muscle cells maintain their NPCs by the removal of whole NPCs from the NE in an ESCRT dependent manner, which are subsequently replaced by newly assembled NPCs 67. Terminally differentiated muscle cells maintain their NPCs by piecemeal replacement of subunits, resulting in NPC composed of Nups with different ages 67. Future studies will have to resolve whether the mechanisms described for quality control during NPC assembly in yeast 68, 69 could also be used to identify damaged NPCs in postmitotic cells. Alternatively, damaged NPCs might be cleared as a whole through autophagy, specifically through microautophagy at nuclear vacuolar junctions or by selective autophagy. The possibility that damaged NPCs are not cleared from the NE at all in some cell types cannot be ruled out either. The fact that at least two NPC maintenance mechanisms exist, combined with the possibility that different cells favor different mechanisms of NPC maintenance, will contribute to the age‐related differences in Nup abundance across different model organisms and/or organ tissues. Other outstanding questions are how damaged NPCs are sensed, how cells control which NPCs or NPC subunits are replaced, and what causes NPCs to become damaged in time.

### What could be sources of damage to NPCs in aging?

The most frequent source of protein damage considered in aging is oxidative damage 106. Replicative and chronologically aged cells of diverse origins have higher levels of reactive oxygen species (ROS) than at young age, and show signs of oxidative stress 107, 108. Moreover, levels of carbonylated proteins, which are caused by ROS, are increased in aged cells 109. In yeast, carbonylated proteins are retained by the mother cell during division 110 contributing to her aging.

Oxidation of the amino acid side chains of lysine, arginine, proline, and threonine causes these side chains to be replaced by carbonyl groups. Carbonylation of those amino acids is irreversible and consequently, carbonylated proteins are altered in their charge and hydrophobicity impacting their synthesis, stability, and functionality 111. On the other hand, ROS are also important intracellular signaling molecules that can trigger protective responses 112, 113, 114. The main source of intracellular ROS stems from mitochondrial respiration 114. Other sources of intracellular ROS include NAD(P)H oxidases at the plasma membrane, peroxisomes, D‐amino acid oxidases in the cytoplasm, and disulfide bond formation at the ER 114, 115. In yeast, the NADH oxidase orthologue Yno1 additionally contributes to ROS formation at the ER/NE network 116 and may be closest to the NPCs. So, while it is clear that ROS and carbonylation contribute to age‐dependent changes of cellular systems, should we expect the NPC to be vulnerable to oxidative damage and if so, how would this affect nucleocytoplasmic transport?

D'Angelo *et al*. show in their 2009 paper that carbonyl groups can be detected on Nup93 and Nup153, but not on Nup107 isolated from old rat brains. The proteins that form the scaffold of the NPC might thus be somewhat protected against oxidative damage. Carbonylation of the long‐lived linker Nup93 might especially reduce the structural integrity of the NPC which will cause NPCs to become more leaky with aging 4, 5, 6, 16, 17, 117. A direct effect of carbonyl modification of the FG‐Nups on the permeability of NPCs is less likely, as FG‐Nups do not readily oxidize 11. Moreover, based on coarse‐grained molecular dynamics simulations 118, carbonyl modified FG‐Nups show little conformational changes compared to FG‐Nups without carbonyl modifications, suggesting that protein carbonylation of FG‐Nups has only a minor impact on the permeability of the NPC 11. Apart from the impact that oxidative stress may have on the structure of the NPC, there are effects on the Ran‐GDP/GTP gradient in the cell 119 that cause nucleocytoplasmic transport rates to decrease under oxidative stress. Altogether, oxidative stress impacts nuclear transport 119 and carbonylation of Nup93 might reduce the structural integrity of NPCs in aged cells, but direct carbonylation of FG‐Nups is not likely to play a role in aging.

Instead, we suggest that the unique interior of the NPC, with ultra‐high concentrations of the disordered FG‐Nups, should be considered in the context of aging. The FG‐Nups may be at risk for aggregation in aging as IDPs, including FG‐Nups, are known to be aggregation‐prone 66, 120 and protein aggregation in general increases during aging 121, 122. IDPs, like the FG‐Nups, do not have a stable secondary or tertiary structure. Instead, they exist in a large set of readily interchangeable conformations. While we know well how cells guard the structure of stably folded proteins 123, 124, 125, 126, 127, we know virtually nothing about the mechanisms that guard IDPs. IDPs, including IDPs related to degenerative diseases such as Huntington's disease and amyloid lateral sclerosis, and FG‐Nups, can phase separate to form liquid‐liquid demixed droplets 128 or hydrogels 129, 130, 131 or aggregate to form amyloid fibers 64, 65, 66, 132, 133, 134, 135, 136.

During transport, NTRs modulate the biophysical state of the disordered FG‐Nups inside the NPC by engaging in rapid binding and unbinding events 22, 137, 138, 139, 140. Recent data suggest that NTRs can also modulate the biophysical state and toxicity of several IDPs related to neurodegenerative diseases 134, 141, 142, 143, 144, 145, 146. Moreover, several repeat‐proteins that are associated with neurodegenerative diseases are known to disrupt nucleocytoplasmic transport 34, 136, 142, 144, 146, 147, 148, 149. Altogether, these recent findings suggest that the FG‐Nups may be at risk in aging if NTR levels become limiting, or if cells have a larger load of aggregation‐prone proteins that may sequester NPC components into aberrant phase‐separated states or aggregates. A full understanding of the stability of the disordered phase in normal aging is not available at present, but considering the tight connection with aggregation pathologies and NPCs, we consider this a valuable research area for the future.

### Outlook

In this review, we discuss that age‐related changes at the NPC and nucleocytoplasmic transport are diverse in different tissues and model organisms and we have tried to find potential reasons why the conserved aging process might be so diverse at the level of the NPC. We explain how NPC assembly might be challenging for replicative aging cells and that NPC maintenance might be challenging for chronologically aging cells. In aging tissues, a mix of both will be observed depending on the regenerative capacity of the tissue. In addition, there are two different NPC assembly mechanisms, and at least two different mechanisms of NPC maintenance, adding another explanation why age‐related changes at the NPC might be diverse in different cells. NPC interphase assembly and NPC maintenance mechanisms are currently least understood. In this context, the ESCRT‐III system is particularly interesting to study because it is involved in NPC assembly 69 and NPC maintenance 67. Age‐dependent changes in ESCRT function could, therefore, impact NPC assembly in aging dividing cells and NPC maintenance in chronological aging cells. At least in replicative aging yeast, ESCRT function is likely to be compromised by the drastically reduced abundance of Vps4 and reduced levels of Heh2 at the NE 11, but virtually nothing is known about ESCRT function during aging in other replicative aging cells.

Nucleocytoplasmic transport is influenced by the structure and numbers of NPCs, but also by the abundance of NTRs and the Ran‐GDP/GTP gradient. We consider it likely that changes in nucleocytoplasmic transport or passive diffusion will cause changes in the localization of proteins during aging. The overall changes in nucleocytoplasmic transport might be moderate because a full complement of nonfunctional NPCs is not compatible with life. More research is needed to understand how the nucleocytoplasmic transport network operates and how changes in protein localization influence the aging process. It remains to be established if a widespread mislocalization of nuclear proteins could be causal to the loss of protein homeostasis observed in many aggregation pathologies.

The cell also uses NPCs as an anchoring point for various processes, which may alter when the architecture or number of NPCs changes in aging cells. For example, NPCs play an important role in genome stability and organization 150: they are used as sites of gene activation 151, 152, 153 [reviewed in 147], for anchoring of eroded telomeres, ERCs 154, and repair of DNA double‐strand breaks 155, 156, 157. More speculative, changes in NPC number and architecture could also change local protein degradation by the NPC‐localized proteasome 158, 159 or local translation of NPC components 160. We conclude that nuclear pores, the physical gatekeepers to the nuclear interior, may well represent an important gatekeeper in aging, and boosting its quality control may provide opportunities to increase resilience to aging and age‐related diseases.

## Conflicts of interest

The authors declare no conflict of interest.

## Author contributions

ILR, AS, and LMV wrote the manuscript.
